# Narcissism, Social Encounters, and Mood in Late Life

**DOI:** 10.1093/geroni/igaa057.1249

**Published:** 2020-12-16

**Authors:** Shiyang Zhang, Yee To Ng, Karen Fingerman

**Affiliations:** The University of Texas at Austin, Austin, Texas, United States

## Abstract

Social contacts have a strong impact on older adult’s well-being. However, narcissism (i.e., feelings of self-importance) may undermine interpersonal connections. Research with young adults has found that being narcissistic may generate feelings of boredom, irritation, and pride because narcissistic young adults oftentimes have difficulty maintaining attention, have greater sensitivity to negative social events (e.g. social rejections), and have an exaggerated sense of self-worth. Yet, we know little about narcissism in late life, particularly in a daily context. This study examined the associations between narcissism, social encounters, and mood (i.e., bored, irritated, lonely, proud) throughout the day. Older adults aged 65 + (N = 307) from the Daily Experiences and Well-being Study completed a measure of narcissism in a baseline interview. Then, in ecological momentary assessments (EMA), they reported social encounters and mood every 3 hours for 5 to 6 days. We found no significant associations between narcissism and number of social encounters throughout the day. Multilevel models revealed that older adults who scored higher on narcissism felt more bored and prouder throughout the day. Interaction terms involving narcissism and social encounters showed that during assessment periods when they had social encounters, participants who scored higher on narcissism reported a similar level of loneliness as when they were alone, whereas their peers who scored lower on narcissism experienced decreases in loneliness. Findings suggest that narcissism does not predict social encounters. However, older adults who are higher in narcissism may be less likely to be influenced by their social encounters.

